# Diagnostic Performance of Salivary PCR for the Detection of Congenital Cytomegalovirus: A Systematic Review and Meta-Analysis

**DOI:** 10.3390/v17091253

**Published:** 2025-09-17

**Authors:** Sara Mohammed Ahmed Rady, Mahmoud Ibrahim Abdelmoati, Sara Sabra, Maryam Alameddine, Suchita Dsilva, Jeevan K. Shetty

**Affiliations:** 1 School of Medicine, Royal College of Surgeons in Ireland-Medical University of Bahrain (RCSI-MUB), Busaiteen P.O. Box 15503, Bahrain; 22200800@rcsi-mub.com (S.M.A.R.); 21200683@rcsi-mub.com (M.I.A.); 22200646@rcsi-mub.com (S.S.); 22200492@rcsi-mub.com (M.A.); sdsilva@rcsi-mub.com (S.D.); 2 Department of Library & Learning Resource Centre (LLRC), Royal College of Surgeons in Ireland-Medical University of Bahrain (RCSI-MUB), Busaiteen P.O. Box 15503, Bahrain; 3 Department of Biochemistry, Royal College of Surgeons in Ireland-Medical University of Bahrain (RCSI-MUB), Busaiteen P.O. Box 15503, Bahrain

**Keywords:** congenital cytomegalovirus, salivary PCR, neonatal screening, diagnostic accuracy, systematic review, meta-analysis, sensitivity and specificity, non-invasive testing, early detection

## Abstract

Congenital cytomegalovirus (CMV) is a major cause of neonatal morbidity, particularly sensorineural hearing loss, yet its early detection remains challenging. While urinary PCR is the current diagnostic gold standard, its implementation in neonatal settings is often limited by feasibility issues. Salivary PCR presents a more practical alternative, but its diagnostic accuracy has remained uncertain. This systematic review and meta-analysis aimed to evaluate the performance of salivary PCR compared to urinary PCR in detecting congenital CMV in neonates. Following PRISMA guidelines, 15 observational studies involving 29,617 neonates were analyzed using a random-effects model. Pooled sensitivity and specificity were 0.99 and 1.00, respectively, with a negative predictive value (NPV) of 1.00 and a positive predictive value (PPV) of 0.91, despite moderate heterogeneity. Subgroup analysis showed high diagnostic performance across general neonates, infants of seropositive mothers and high-risk neonates (referring to neonates that are small for their gestational age (SGA), neonates who failed hearing screening, and neonates with CMV-related congenital abnormalities). The general group had the highest specificity (0.999), while high-risk neonates showed the highest sensitivity (0.981). Across all groups, NPV remained consistently above 0.994, with PPV ranging from 0.848 to 0.981. These findings confirm that salivary PCR is a highly accurate and feasible tool for congenital CMV diagnosis.

## 1. Introduction

Cytomegalovirus (CMV) is a double-stranded DNA virus and a member of the *Herpesviridae* family [1]. The human cytomegalovirus virion consists of an icosahedral capsid with attached proteins, an outer layer of proteins called the tegument, and a surrounding lipid envelope containing viral glycoproteins [1]. This pathogen infects individuals of all ages and targets a wide range of tissues including blood, brain, and heart [1]. It has high prevalence worldwide, affecting half the population in developed countries and almost 100% in developing countries, particularly in Asia and Africa [2,3].

CMV spreads through various transmission routes, primarily via direct contact with infected body fluids such as urine, saliva, and blood [4]. Additionally, a notable transmission route is during pregnancy, in which the virus can pass through the placenta, causing congenital CMV in the neonate [4]. Once a person is infected, CMV establishes lifelong latency by persisting in myeloid cells [4]. While the virus often remains dormant in healthy individuals, it has the potential to reactivate when the immune system is weakened. During reactivation, CMV remains largely cell-associated in the bloodstream [5]. Dissemination occurs primarily through circulating infected leukocytes (e.g., monocytes, polymorphonuclear cells) and endothelial cells [5]. This cell-associated viremia facilitates viral spread to multiple organ systems [5]. This ability to remain latent and reactivate under certain conditions makes CMV a major concern, particularly for immunocompromised individuals and newborns.

According to the CDC, approximately 1 in 200 babies are born with congenital CMV, and a staggering 1 in 5 suffer from birth defects or long-term ailments [6]. Around 10 to 15% of asymptomatic newborns lose their hearing later in life [7]. Hearing loss can worsen from mild to severe within the first two years of life, which is a crucial period for language development; over time, it affects the child’s ability to develop communication, language, and social skills, which causes cognitive impairment [8]. Infected patients could also develop vestibular disorders, leading to balance and motor deficits, among other severe complications, including microcephaly, intracranial calcifications, and cerebral hypoplasia [9]. Therefore, timely diagnosis and intervention for congenital CMV in neonates are crucial to avoid such complications.

The cost of illness for congenital CMV was estimated at $4 billion in the USA (2001) and £732 million in the UK (2016), highlighting an example of its substantial economic burden [10,11].

Traditionally, urine polymerase chain reaction (PCR) has been considered the gold-standard method of diagnosis for congenital CMV, but it faces challenges in time and collection [12,13]. A prospective study described it as “prohibitive”, energy-intensive, requires frequent bag changes, is prone to contamination by meconium and leakage, all of which led to sample loss and delay in testing. This can prevent discrimination between acquired and congenital CMV [4,6,13,14].

Given these challenges, saliva PCR has emerged as a viable alternative due to its feasibility. Infants with congenital CMV shed significant quantities of the virus in their saliva, making it a useful screening technique [15]. Saliva specimens are collected through cheek swabs from neonates and sent for PCR [15]. Delays in sampling can subject this mode to false positive congenital CMV, as CMV may be present in the breastmilk of seropositive mothers [16]. However, compared to urine samples, saliva remains are less prone to contamination and more easily collected [17].

Despite the promise of salivary PCR, the existing literature is limited in depth and scope. Therefore, the aim of this study is to assess the diagnostic test accuracy of salivary PCR as an alternative to urinary PCR in effectively detecting congenital CMV. This study aims to thoroughly explore diagnostic metrics, including specificity (SP), sensitivity (SN), Positive Predictive value (PPV), and Negative Predictive Value (NPV). Given the surge in scientific research, an updated meta-analysis is warranted to capture new global data. Also, this study will focus exclusively on the neonatal population, which is often underrepresented in similar reviews. Finally, reducing heterogeneity to strengthen the validity of conclusions will be a key priority.

## 2. Materials and Methods

### 2.1. Protocol and Registration

The protocol for this systematic review and meta-analysis was developed in accordance with the Preferred Reporting Items for Systematic Review and Meta-Analysis Protocols (PRISMA) 2020 guidelines to ensure methodological transparency and rigor [18]. The protocol was prospectively registered in the PROSPERO database under the registration number CRD42024603785 [19].

### 2.2. Search Protocol

A literature search was comprehensively performed across eight electronic databases: PubMed, Scopus, Web of Science, ScienceDirect, ProQuest, Cochrane Library, CINAHL, and Wiley. The search strategy was developed using Medical Subject Headings (MeSH) terms and relevant keywords (Appendix A).

### 2.3. Study Selection

Study selection was performed in three phases using Covidence, a systematic review management tool. First was the removal of duplicates, which was conducted mainly by Covidence automatically. Secondly, for title and abstract screening, two independent reviewers screened all studies based on the following specified inclusion and exclusion criteria in Table 1, and conflicts were resolved by another two independent reviewers. Subsequently, the same process was followed for full-text screening, but by the opposite set of reviewers.

### 2.4. Quality Assessment

Risk of Bias was assessed using the **QUDAS-2 tool** (Quality Assessment of Diagnostic Accuracy Studies) as recommended by the Cochrane Collaboration [20]. QUADAS-2 assesses four key domains: patient selection, index test, reference standard, and flow and timing.

Studies were categorized as having low, high, or unclear risk of bias or applicability concern in each domain, based on standardized questions (Appendix B, Table A1). The assessment was performed independently by two reviewers, with conflicts resolved through group discussion. This ensured a transparent and unbiased quality assessment to determine the overall strength of the evidence.

### 2.5. Data Extraction

**Figure A1:**
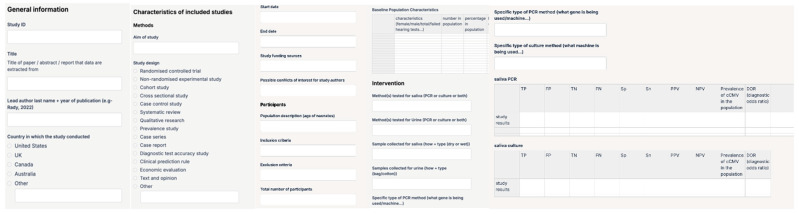
Data Extract Template on Covidence.

For each study, relevant data were extracted, including the total number of participants, sample collection method, and population type, among other variables. When diagnostic accuracy metrics such as SN, SP, PPV, and NPV were reported, they were directly extracted. In cases where these metrics were not available, raw values for true positives (TP), true negatives (TN), false positives (FP), and false negatives (FN) were extracted to enable calculation. All data were systematically recorded and managed using a standardized template within Covidence, as presented in Appendix C, Figure A1.

### 2.6. Missing Data Calculations

In this study, missing proportions were calculated from extracted raw data using the Proportions were calculated using the **Binomial Proportion equation**:

**p^ = x/n,** (1)

where p^ is the estimated proportion (e.g., sensitivity, specificity, PPV, or NPV), x is the number of successes (e.g., true positives for sensitivity), and n is the relevant sample size. This equation forms the basis for all diagnostic performance metrics presented in Equation (2).

Diagnostic accuracy measures were calculated as follows:

**SP = TN/(TN + FP), SN = TP/(TP + FN), PPV = TP/(TP + FP), NPV = TN/(TN + FN),** (2)

where **TP** = true positives, **TN** = true negatives, **FP** = false positives, and **FN** = false negatives.

The standard error for each proportion was calculated using the Delta Method (Equation (3)) to account for variability in Wilson-adjusted proportions [21]:

**SE_Wilson_ = √[p^(1 − p^)/n + z^2^/(4n^2^)],** (3)

where *z* is the z-score for the desired confidence level (*z* = 1.96 for 95% confidence), and *n* is the sample size.

The 95% confidence interval (CI) was calculated using the Wilson Score Interval without Continuity Correction:

**CI = (p^ + z^2^/(2n) ± z × √[p^(1 − p^)/n + z^2^/(4n^2^)])/(1 + z^2^/n),** (4)

which provides more accurate interval estimates in small or extreme samples [21]. This approach improves the robustness of our estimates and aligns with best practices in diagnostic accuracy studies.

### 2.7. Data Analysis

The meta-analysis was conducted using R software (version 4.4.3) with the ‘meta’ [22] and ‘metafor’ [23] packages to evaluate the diagnostic performance of salivary PCR compared to urinary PCR in detecting congenital CMV. A random-effects model employing the DerSimonian and Laird method was used to calculate pooled estimates of diagnostic accuracy, including SN, SP, PPV, and NPV; this model accounts for anticipated heterogeneity among studies [24].

Expanded diagnostic metrics were also calculated using the same method, which include Diagnostic Odds Ratio (DOR), Area Under the Curve (AUC), Positive Likelihood Ratio (PLR), and Negative Likelihood Ratio (NLR).

A Summary Receiver Operating Characteristic (SROC) Curve was made to visually represent the overall diagnostic performance of Salivary PCR. A funnel plot was then constructed to evaluate small-study effects and potential publication bias, which was further assessed using Egger’s test.

Then, a subgroup analysis was conducted across three clinically relevant neonatal populations: All neonates, neonates of seropositive mothers, and, lastly, high-risk neonates, which generally refers to the following three characteristics: neonates that are SGA, neonates who failed hearing screening, and neonates with CMV-related congenital abnormalities. Pooled diagnostic metrics were calculated and presented separately for each subgroup using random-effects forest plots. Additionally, the same expanded diagnostic metrics were calculated for each group. This allowed for comparative assessment of salivary PCR performance across different population contexts

## 3. Results

### 3.1. Screening Process

**Figure 1:**
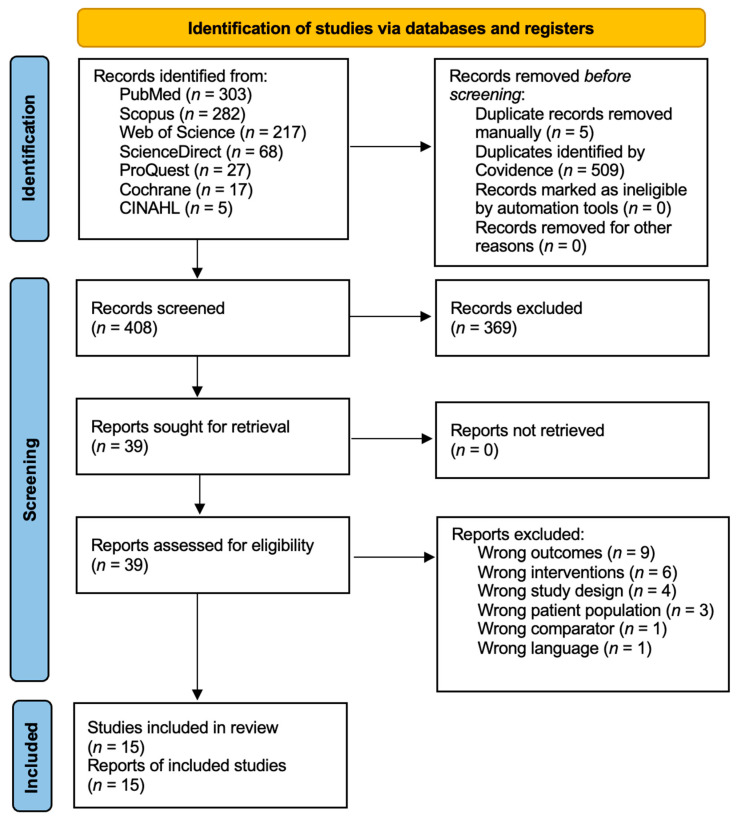
PRIMA flow diagram.

A total of 922 identified studies were explored from the following databases: 303 from PubMed, 282 from Scopus, 217 from Web of Science, 68 from ScienceDirect, 27 from ProQuest, 17 from Cochrane, 5 from CINAHL, and 3 from Wiley. However, only 408 studies were eligible for screening since 514 duplicates were excluded. A total of 369 studies were excluded during the abstract screening process, leaving 39 full-text papers for detailed evaluation. Among these, 9 were excluded for wrong outcomes, 6 for wrong interventions, 4 for wrong study design, 3 for wrong patient population, 1 for wrong comparator and finally 1 for wrong language, all of which amounted to 23 studies. A final 15 papers were selected for further data extraction. Refer to Figure 1 for more details.

### 3.2. Quality Assessment Results

**Figure 2:**
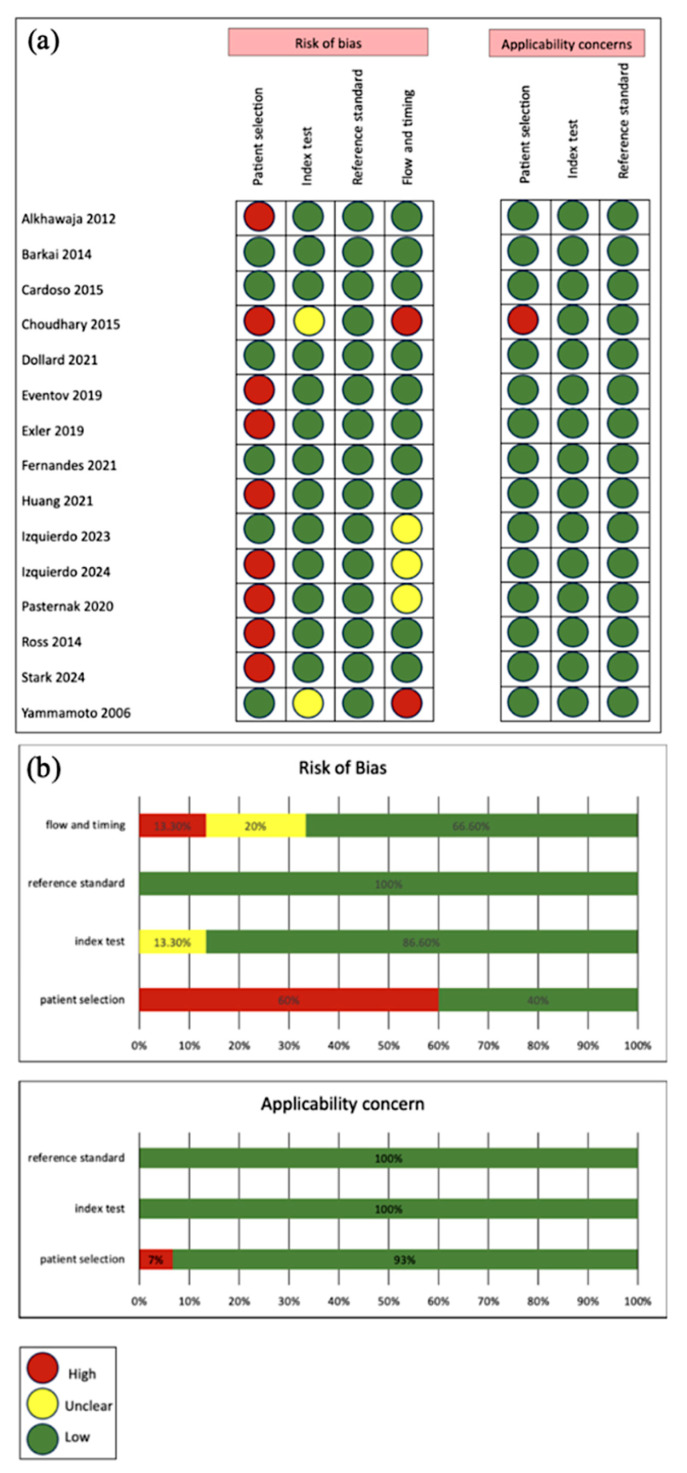
(**a**) Study-level QUADAS-2 risk of bias and applicability assessments across domains [17,25,26,27,28,29,30,31,32,33,34,35,36,37,38]; (**b**) Aggregated domain-level percentages for risk of bias and applicability concern.

Figure 2 presents the quality assessment of the included studies using the QUADAS-2 tool. Overall, studies showed low risk of bias and low applicability concern, supported by clearly described methods. However, some studies had high risk of bias due to discriminatory patient selection: 8 studies tested patients that are high risk, suspected or confirmed to have CMV. Studies marked as unclear lacked sufficient information for a definitive judgment.

### 3.3. Study Characteristics

The raw data tables are present in Appendix D, Table A2. From the 15 included studies [17,25,26,27,28,29,30,31,32,33,34,35,36,37,38] we extracted 3 additional datasets; one from Izquierdo (2024) and two from Pasternak (2020); giving a total of 18 datasets. Specificity ranged from [0.904–1.000], sensitivity [0.245–1.000], PPV [0.456–1.000], and NPV [0.976 to 1.000]. Two anomalous studies were manually identified, calling for a Leave-One-Out (LOO) analysis to assess their impact. Most studies were prospective cohort studies conducted in the Americas WHO region. Study characteristics are detailed in Appendix E, Table A3. Three populations were represented: high-risk neonates, infants of seropositive mothers, and all neonates, with high-risk neonates being the most common.

### 3.4. Exclusion of Outliers

A Leave One Out (LOO) analysis was performed, identifying two studies contributing significantly to heterogeneity (Appendix F, Table A4, Table A5, Table A6 and Table A7).Huang 2021 was excluded in NPV and SN analysis as the estimates were notably higher, 0.99998 and 0.9875, respectively, after its exclusion [33]. Additionally, when excluded, the Tau^2^ and I^2^ values dropped to zero, confirming their role in heterogeneity.

Eventov-Friedman 2019 was excluded in PPV and SP analysis as it also gave higher estimates, 0.9078 and 0.99908, respectively, when excluded [29]. Moderate heterogeneity in PPV and SP (I^2^ = 51.49%, 57.23%) was significantly reduced upon exclusion, indicating its disproportionate influence on pooled PPV and SP results.

Table 2 and Table 3 are sample LOO tables showing an example of the significant changes with the exclusion of the previously mentioned studies in comparison to Dollard 2021 [17] as a control exclusion.

### 3.5. Meta-Analysis of Diagnostic Test Accuracy

#### 3.5.1. Random Effects Forest Plots for All Studies

**Figure 3:**
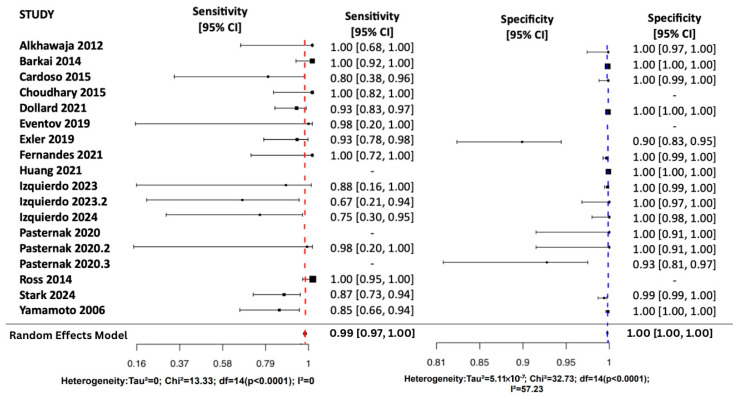
Random Effects Forest Plots for SN and SP [17,25,26,27,28,29,30,31,32,33,34,35,36,37,38].

**Figure 4:**
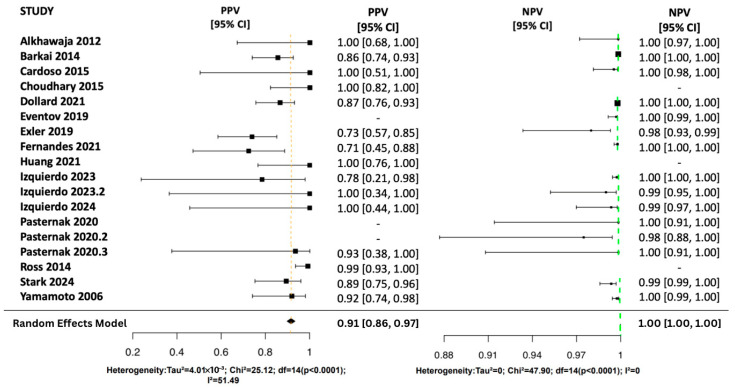
Random Effects Forest Plots for PPV and NPV [17,25,26,27,28,29,30,31,32,33,34,35,36,37,38].

Using a Random Effects Model, the review assessed all four diagnostic metrics shown in Figure 3 and Figure 4. Sensitivity pooled at 0.99 (95% CI: [0.97, 1.00]), showing excellent detection of true positives. Most studies reported sensitivity above 0.85, and heterogeneity was low (I^2^ = 0%) suggesting low variability among studies. Specificity pooled at 1.00 (95% CI: [1.00, 1.00]), indicating a very low false-positive rate. Although most studies reported values near 1.00, moderate heterogeneity (I^2^ = 57.2%) suggests some study-level variation.

The pooled NPV was 1.00 (95% CI: [1.00, 1.00]), indicating a strong ability to rule out disease with low variability (I^2^ = 0%). Individual NPVs ranged from 0.98 to 1.00 with narrow confidence intervals. The pooled PPV was 0.91 (95% CI: [0.86, 0.97]), reflecting a high likelihood of correctly identifying disease. However, PPV varied across studies (0.71–1.00), with moderate heterogeneity (I^2^ = 51.5%).

#### 3.5.2. Expanded Diagnostic Metrics for All Studies

Table 4 displays the summary meta-analysis results for all the studies. The DOR of 2041.14 (95% CI: 778.995–5348.226) reflects a very high level of overall test accuracy, indicating strong separation between those with and without the disease. The AUC of 0.99 (95% CI: 0.97–1.00) further supports this, showing that salivary PCR has excellent discriminatory ability, with near-perfect sensitivity and specificity.

A high PLR of 109.87 [33.78–357.39] suggests that a positive result strongly confirms the presence of the disease. Meanwhile, the low NLR of 0.0885 [0.0409–0.1916] indicates that a negative result effectively rules it out, highlighting the test’s strong diagnostic accuracy.

#### 3.5.3. The Summary Receiver Operating Characteristic (SROC) Curve

**Figure 5:**
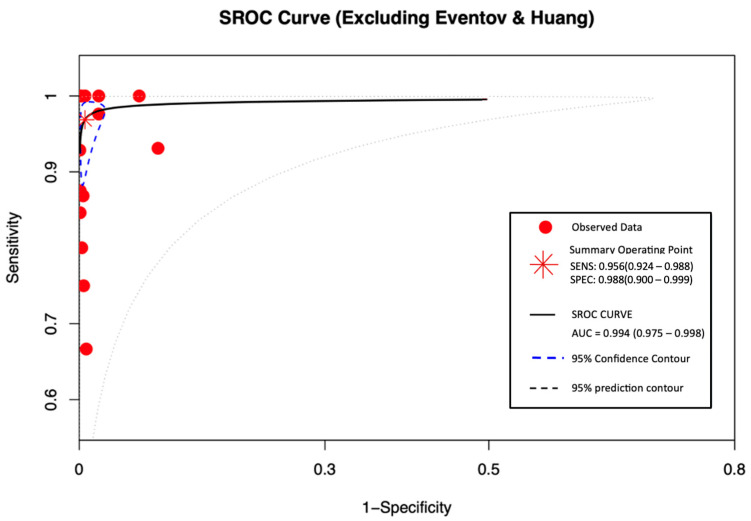
SROC curve excluding Eventov and Huang.

SROC curve assesses overall diagnostic accuracy by plotting sensitivity against specificity (False-positive rate) as shown in Figure 5. All 16 datasets were included. The sensitivity was 0.956 (95% CI: 0.924–0.988), and the specificity was 0.988 (95% CI: 0.900–0.999). The AUC was 0.994 (0.975–0.998), reflecting excellent discriminatory power with minimal misclassification. These findings show that the diagnostic test demonstrated high performance.

#### 3.5.4. Publication Bias

**Figure 6:**
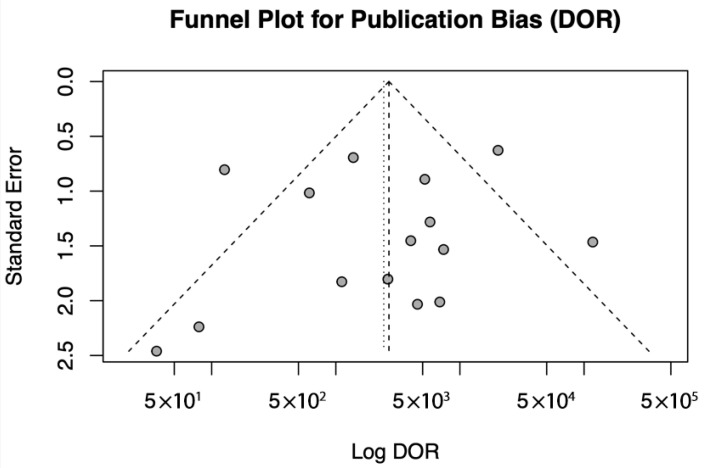
Funnel Plot for Publication Bias.

This meta-analysis confirms that the diagnostic test exhibits high sensitivity, specificity, and predictive accuracy across different populations. However, publication bias and study-level influences were evaluated using Egger’s test and the funnel plot to ensure the robustness of these findings as shown in Figure 6 and Table 5.

A funnel plot of log DOR against standard error was generated to assess publication bias. Individual studies are shown as points, and the pooled effect is represented by a vertical dotted line. The dashed lines correspond to the expected 95% confidence region in the absence of bias.

In the assessment of publication bias, a funnel plot was generated for DOR, and Egger’s test was performed to statistically evaluate asymmetry. The funnel plot exhibited some visual asymmetry; however, Egger’s test did not provide significant evidence of publication bias (t = −0.4149, df = 13, *p* = 0.685). The estimated effect size when the standard error approached zero was 8.3678 (95% CI: 5.6490–11.0866), indicating a stable overall effect. These findings suggest that any observed asymmetry is likely due to random variation or small-study effects rather than systematic publication bias.

### 3.6. Meta-Analysis of Diagnostic Test Acuuracy of Subgroups

#### 3.6.1. SN and SP Random Effects Forest Plots for Subgroups

**Figure 7:**
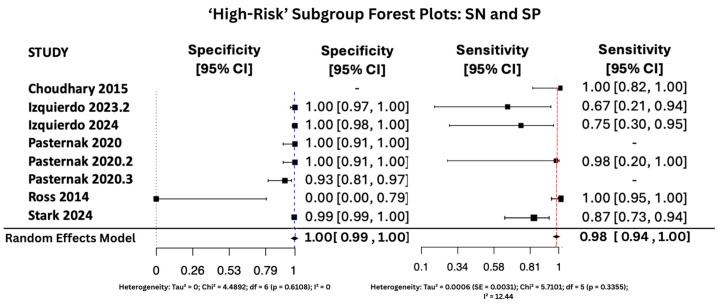
SP and SN Forest Plots for ‘High-Risk’ Subgroup [15,28,34,35,36,37]. The red dashed vertical line corresponds to the pooled sensitivity estimate, while the blue dashed vertical line denotes the pooled specificity estimate.

**Figure 8:**
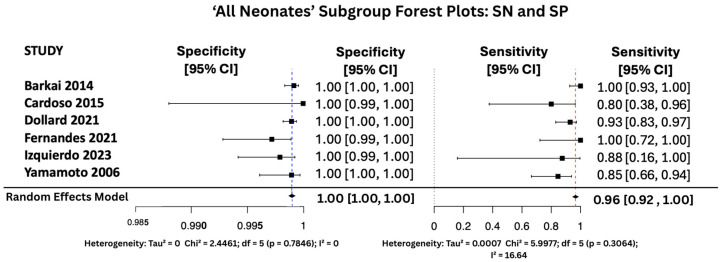
SP and SN Forest Plots for ‘All Neonates’ Subgroup [17,26,27,31,34,38].

**Figure 9:**
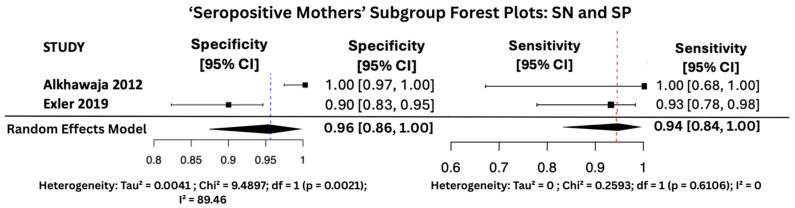
SP and SN Forest Plots for ‘Seropositive Mothers’ Subgroup [25,30].

Figure 7, Figure 8 and Figure 9 display the forest plots of sensitivity and specificity for salivary PCR in each subgroup. Specificity was nearly perfect in all neonate subgroups at 0.9990 (95% CI: 0.9986–0.9994) and high-risk populations at 0.9956 (95% CI: 0.9909–1.00), but notably lower in the seropositive mother subgroup at 0.9566 (95% CI: 0.8629–1.00). Heterogeneity in specificity was only significant in the seropositive group (I^2^ = 89.46%).

Sensitivity was also consistently high, with the highest observed in the high-risk group at 0.9810 (95% CI: 0.9353–1.00), followed by 0.9648 (95% CI: 0.9163–1.00) in All neonates group, and 0.9436 (95% CI: 0.8410–1.00) in seropositive mothers. Heterogeneity for sensitivity was low across all groups, with I^2^ values ranging from 0.00% to 16.64%, indicating good consistency between studies.

#### 3.6.2. PPV and NPV Random Effects Forest Plots for Subgroups

**Figure 10:**
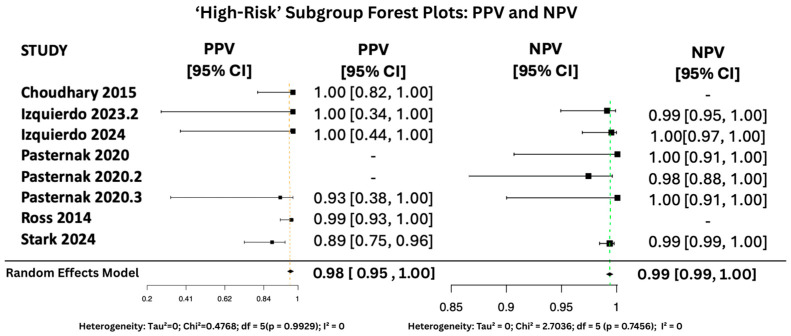
PPV and NPV Forest Plots for ‘High-Risk’ Subgroup [15,28,34,35,36,37]. The yellow dashed vertical line corresponds to the pooled PPV estimate, while the green dashed vertical line denotes the pooled NPV estimate.

**Figure 11:**
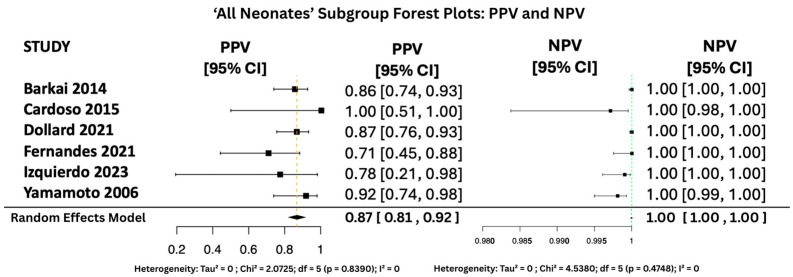
PPV and NPV Forest Plots for ‘All Neonates’ Subgroup [17,26,27,31,34,38].

**Figure 12:**
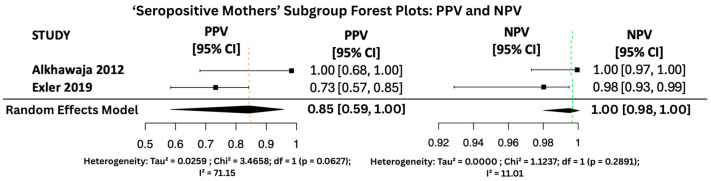
PPV and NPV Forest Plots for ‘Seropositive Mothers’ Subgroup [25,30].

Figure 10, Figure 11 and Figure 12 display the forest plots of PPV and NPV for salivary PCR in each subgroup. In terms of predictive values, the Positive Predictive Value (PPV) was highest in the high-risk population (0.9817, 95% CI: 0.9507–1.00), moderate in All neonates group (0.8674, 95% CI: 0.8092–0.925), and lowest in the seropositive group (0.8484, 95% CI: 0.5857–1.00). PPV heterogeneity was moderate in the seropositive mothers (I^2^ = 71.15%) but negligible in the other two subgroups.

The Negative Predictive Value (NPV) was consistently very high across all groups, with values of 0.99999 (95% CI: 0.9998–1.00) in All neonates group, 0.9965 (95% CI: 0.9816–1.00) in seropositive mothers, and 0.9940 (95% CI: 0.9889–0.9991) in high-risk individuals. Heterogeneity for NPV was minimal to none (I^2^ = 0–11%).

#### 3.6.3. Expanded Diagnostic Metrics for Subgroups

Table 6 displays the summary meta-analysis results for all the subgroups. The diagnostic performance was highest in the general neonate population, with a DOR of 8635.79 (95% CI: 3197.51–23,323.41), compared to 897.15 (95% CI: 351.84–2287.65) in high-risk populations and 437.03 (95% CI: 10.75–17,754.31) in seropositive mothers.

AUC was similarly high across all subgroups, reflecting excellent overall test performance: 0.9818 (95% CI: 0.9575–1.00) in all neonate groups, 0.9424 (95% CI: 0.8681–1.00) in seropositive mothers, and 0.9259 (95% CI: 0.8681–0.9836) in high-risk populations.

PLR was markedly highest in the All-neonate population at 833.01 (95% CI: 517.11–135.20), followed by 37.10 (95% CI: 1.416–971.59) in seropositive mothers, and 29.49 (95% CI: 6.43–135.20) in high-risk groups.

Finally, NLR was lowest in the seropositive mother group (0.0849, 95% CI: 0.0270–0.2667) and remained similarly low in the high-risk group (0.0914, 95% CI: 0.0382–0.2188). In the general neonatal group, the NLR was slightly higher at 0.1141 (95% CI: 0.0620–0.2099), yet still within a range suggestive of good diagnostic performance.

## 4. Discussion

### 4.1. All Studies Discussion

Findings from the 15 studies [17,25,26,27,28,29,30,31,32,33,34,35,36,37,38] confirm excellent diagnostic performance, with high NPV (99.99%), SN (98.75%), and SP (99.91%), indicating low false-negative and false-positive rates. A meta-analysis by Zheng et al. (2022), conducted on all infants rather than neonates alone, supports these results, reporting 100% SP and 97% SN [39].

Additionally, authoritative sources from the CDC, Cleveland Clinic and Boston Children’s Hospital all recognize salivary PCR as a diagnostic method for congenital CMV further supporting the reliability of salivary PCR’s diagnostic ability [40,41,42].

The pooled PPV is 90.78% ([95% CI: 86.00–96.28]), with variability likely driven by differences in population prevalence (high-risk neonates, infants of seropositive mothers, and all neonate populations). Biologically, this is supported by the shedding of CMV in the breast milk of seropositive mothers, which may lead to transient viral presence in the infant’s saliva and potential false-positive results [40]. This reflects postnatal exposure rather than congenital infection, making this distinction essential for accurate interpretation of positive saliva PCR results.

To address PPV variability, likelihood ratios were assessed, with PLR > 10 (109.87 [95% CI: 33.77–357.39]) and NLR < 0.1 (0.088 [95% CI: 0.0409–0.1916]) indicating strong diagnostic performance of salivary PCR in confirming and excluding congenital CMV cases [43]. Similarly, Zheng et al. (2022) reported an exceptionally high PLR of 797.5 and an NLR of 0.09 [39].

This study’s SROC curve demonstrates a high TP rate of 0.956 (95% CI: 0.924–0.988) and low FP rate with a SP of 0.988 (95% CI: 0.900–0.999), along with an AUC of 0.994. This confirms near-perfect discriminatory ability for salivary PCR, supporting the CDC’s decision to use it as one of the diagnostic tools as mentioned above [6].

Given its high sensitivity, specificity, and ease of sample collection, salivary PCR also shows promise as a potential tool for early screening of congenital CMV, particularly in asymptomatic neonates. Its non-invasive nature and logistical feasibility make it a strong candidate for integration into large-scale newborn screening programs [44]. However, the clinical and economic implications of routine screening remain to be fully evaluated [45].

In conclusion, the findings of this systematic review and meta-analysis confirm that saliva PCR is a highly efficient diagnostic tool for congenital CMV due to its exceptional performance, supported by comparisons with Zheng et al. (2022) and authoritative sources [6,39,40,41,42]

### 4.2. Subgroup Discussion

This meta-analysis confirms the robust diagnostic performance of salivary PCR testing for congenital CMV detection across multiple neonatal subgroups. Overall, the test demonstrated exceptional accuracy, with pooled sensitivity and specificity approaching perfection, highlighting its utility as a non-invasive tool for early diagnosis of congenital CMV [34,43].

In the general neonate population, salivary PCR showed outstanding performance, with the highest DOR of 8635.78 and an AUC of 0.98, indicating near-perfect diagnostic accuracy. Both sensitivity (96.5%) and specificity (99.9%) were high, with minimal heterogeneity, reflecting excellent consistency across studies and affirming the test’s reliability in diagnosing congenital CMV.

In the high-risk population subgroup, comprising infants with elevated baseline risk due to clinical symptoms of congenital CMV, diagnostic performance remained highly favorable. Sensitivity reached 98.1%, with specificity of 99.6%, and heterogeneity was low across all diagnostic parameters. These findings suggest that salivary PCR is particularly well-suited to high-risk settings, where early and accurate detection is critical for timely intervention and prevention of long-term complications [46].

The seropositive mothers’ subgroup, while also showing promising results (sensitivity 94.4%, specificity 95.7%), included only two datasets. This limited sample size likely explains the observed heterogeneity in specificity (I^2^ = 89.46%) and PPV (I^2^ = 71.15%). Although the results remain encouraging, the small number of studies reduces the statistical power and generalizability of findings within this subgroup.

As such, caution should be exercised in interpreting these results, and further research is needed to validate test performance in seropositive populations, especially given the potential biological variability and differing baseline prevalence of congenital CMV [47].

Across all subgroups, the negative predictive value (NPV) consistently exceeded 99%, and negative likelihood ratios remained exceptionally low (0.08–0.11), indicating that a negative salivary PCR result is highly reliable for excluding congenital CMV infection. Meanwhile, positive likelihood ratios were extremely high, particularly in the general neonate group (PLR = 833.01), underscoring the strong diagnostic confirmation offered by a positive result.

Together, these findings strongly support the use of salivary PCR testing. Its ease of collection [34], high diagnostic performance, and consistency across both general and high-risk populations, make it an ideal candidate for early detection of congenital CMV. Future studies should aim to expand the evidence base in seropositive maternal cohorts to strengthen confidence in targeted diagnostic strategies.

### 4.3. Strengths

During the study scoping, all the search strategies included title and abstract searches for the key words, increasing the cohesion between the different search strategies for each electronic database.

The use of Wilson Score Interval in this study to estimate missing confidence intervals guaranteed greater accuracy than the traditional Wald method, particularly for small/extreme proportions, which were often present in specificities of 0 or 1 [21].

Additionally, the performance of the LOO for all 4 proportions resulted in exclusion of 2 studies from the meta-analysis, which decreased heterogeneity and increased the results’ accuracy and robustness [48].

The statistical power of this meta-analysis of 15 studies, incorporating a total population of 29,617 neonates, is extremely high (1.0 or 100%), indicating a strong ability to detect a true effect. This is primarily driven by the large sample size, with 480 PCR-positive cases and 29,137 PCR-negative cases [49].

The inclusion of an SROC curve in the analysis provided a comprehensive evaluation of diagnostic performance across studies. The resulting AUC of 0.994 demonstrates near-perfect discriminative ability of the salivary PCR test, reinforcing the robustness and clinical relevance of the pooled findings.

The use of funnel plot analysis and Egger’s test strengthened the validity of the meta-analysis by demonstrating no significant publication bias, thereby increasing confidence in the reliability of the pooled estimates.

The variability in data, indicated by Tau^2^ and I^2^, is moderate or low, which makes the effect sizes more distinct and reliable. Another strength of this review is the targeted focus on the neonatal population, addressing a gap in existing literature where this specific group has been underrepresented [48].

Lastly, these findings demonstrate this meta-analysis provides a highly reliable and statistically robust evaluation of salivary PCR as a diagnostic tool for congenital CMV.

### 4.4. Limitations

Although Alkhawaja (2012) included both congenital and perinatal CMV cases, it was retained despite potential inaccuracies in the congenital CMV population due to its small sample size and limited impact on pooled estimates [25].

Baraki (2014), Fernandez (2021), and Izquierdo (2023) retested only a subset of negative salivary PCR results to evaluate screening protocols [26,31,34]. They were included based on subgroup retesting and shared assumptions, though the possibility of undetected false negatives in untested cases remains.

The exclusion of Huang and Eventov-Friedman may have contributed to overestimating salivary PCR performance, as shown in the LOO analysis in Appendix F, Table A4, Table A5, Table A6 and Table A7 [29,33]. Notably, Eventov-Friedman was the only study to collect neonatal samples within the first 10 days of life, which differs from CDC guidelines recommending collection at 2–3 weeks [4,29].

Both studies also collected and tested saliva and urine samples concurrently, unlike most others. These design differences, along with broader population and epidemiological factors, likely contributed to the observed heterogeneity beyond methodological variation alone.

Moderate heterogeneity was observed in PPV (I^2^ = 51.49%) and specificity (I^2^ = 57.23%), likely due to varying prevalence across studies, directly affecting PPV. Therefore, confirmatory testing may be relevant, as recommended by the CDC [40].

Finally, as previously noted, the inclusion of only two studies within the seropositive mother subgroup significantly limits the strength and generalizability of its findings. The small sample size reduces the statistical power and increases the potential for bias, making it difficult to draw definitive conclusions about diagnostic accuracy in this specific population. Additional studies focusing on seropositive cohorts are needed to validate and strengthen the evidence in this subgroup.

### 4.5. Future Directions

Salivary PCR testing offers a promising avenue for enhancing early detection of congenital CMV. Future research should prioritize prospective cohort studies that evaluate not only their cost-effectiveness but also long-term clinical outcomes following early diagnosis, as well as their feasibility across diverse healthcare settings. The development of standardized protocols, particularly for the optimal timing of sample collection while accounting for potential postnatal exposures, will be essential to maximize diagnostic accuracy.

## 5. Conclusions

This systematic review supports the use of salivary PCR as a reliable diagnostic tool for congenital CMV in neonates, demonstrated by its high sensitivity, specificity, and negative predictive value. The strong diagnostic accuracy, coupled with the practicality and non-invasiveness of saliva collection compared to urine, highlights its potential for widespread use in large-scale screening programs. Subgroup analyses suggest consistent effectiveness across various neonatal populations; however, further research is warranted in seropositive mothers to clarify performance in this subgroup. Future studies should also aim to standardize salivary PCR protocols to enhance consistency and global applicability.

## Figures and Tables

**Table 1:** PICO Inclusion and Exclusion Criteria for Primary and Secondary Outcomes.

(**A**) **Primary Outcome**		
**PICO components**	**Inclusion Criteria**	**Exclusion Criteria**
Population (P)	Symptomatic or asymptomatic neonates (≤28 days old) diagnosed with congenital CMV	Individuals who are not neonates (e.g., children, adults); other infectious diseases in neonates, non-human population
Index test (I)	Saliva PCR used for the detection of congenital CMV	**ONLY** other bodily fluids (e.g., blood, amniotic fluid)
Comparator (C)	Gold standard Urine PCR for the detection of congenital CMV	Comparing **ONLY** to other bodily fluids (e.g., blood, amniotic fluid)
Outcome (O)	Studies reporting SN, SP, PPV, NPV	Absence of raw data, including true-positives (TP), true-negatives (TN), false-positives (FP), and false-negatives (FN)
Study Characteristics	Interventional and Observational studies (cohort, case–control, and cross sectional)	Case reports, reviews, editorials, letters, commentaries
	Studies in English	Studies published in languages other than English
(**B**) **Secondary Outcome**		
**PICO components**	**Inclusion Criteria**	**Exclusion Criteria**
Population (P)	Symptomatic or asymptomatic neonates (≤28 days old) diagnosed with congenital CMV	Individuals who are not neonates (e.g., children, adults); other infectious diseases in neonates, and non-human populations
Index test (I)	Saliva PCR used for the detection of congenital CMV in subgroups of all neonates, high-risk neonates, and seropositive mothers	**ONLY** other bodily fluids (e.g., blood, amniotic fluid)
Comparator (C)	Gold standard Urine PCR for the detection of congenital CMV in subgroups of all neonates, high-risk neonates, and seropositive mothers	Comparing **ONLY** to other bodily fluids (e.g., blood, amniotic fluid)
Outcome (O)	Studies reporting SN, SP, PPV, NPV	Absence of raw data, including true-positives (TP), true-negatives (TN), false-positives (FP), and false-negatives (FN)
Study Characteristics	Interventional and Observational studies (cohort, case–control, and cross-sectional)	Case reports, reviews, editorials, letters, commentaries
	Studies in English	Studies published in languages other than English

**Table 2:** Eventov-Friedman 2019 [29] LOO Sample Table.

Diagnostic Metric	Study Excluded	Random Effects Estimate	Standard Error	Tau^2^	I^2^
Specificity	Dollard 2021 [17]	0.99793	0.00089	4.01000 × 10^−6^	85.98820
	**Eventov-Friedman 2019** [29]	**0.99908**	**0.00038**	**5.10888 × 10^−7^**	**57.122866**
Sensitivity	Dollard 2021 [17]	0.86898	0.05233	0.02211	90.08732
	Eventov-Friedman 2019 [29]	0.87584	0.04691	0.01928	90.17723
PPV	Dollard 2021 [17]	0.85318	0.05056	0.02237	84.51254
	**Eventov-Friedman 2019** [29]	**0.90788**	**0.02801**	**0.00401**	**51.49310**
NPV	Dollard 2021 [17]	0.99807	0.00077	3.08688 × 10^−6^	70.59162
	Eventov-Friedman 2019 [29]	0.99930	0.000	2.57413 × 10^−7^	70.77147

**Table 3:** Huang 2021 [33] LOO Sample Table.

Diagnostic Metric	Study Excluded	Random Effects Estimate	Standard Error	Tau^2^	I^2^
Specificity	Dollard 2021 [17]	0.99793	0.00089	4.01000 × 10^−6^	85.98820
	Huang 2021 [33]	0.99759	0.000996	4.98788 × 10^−6^	84.25072
Sensitivity	Dollard 2021 [17]	0.86898	0.05233	0.02211	90.08732
	**Huang 2021** [33]	**0.98751**	**0.00961**	**0**	**0**
PPV	Dollard 2021 [17]	0.85318	0.05056	0.02237	84.51254
	Huang 2021 [33]	0.84242	0.04843	0.02044	84.96496
NPV	Dollard 2021 [17]	0.99807	0.00077	3.08688 × 10^−6^	70.59162
	**Huang 2021** [33]	**0.99999**	**6.12073 × 10^−5^**	**0**	**0**

**Table 4:** Summary Meta-analysis Results.

Outcome Measures	All Studies (Excluding − Eventov + Huang)
Total population	29,617
Total Positives	480
Total Negatives	29,137
Diagnostic Odds Ratio (DOR)	2041.137 [778.9949, 5348.226]
Area Under the Curve (AUC)	0.9943729 [0.9751325, 0.9987089]
Sensitivity (SN)	0.9875060 [0.9518955, 0.9977854]
Specificity (SP)	0.9990828 [0.9983696, 0.9996323]
Positive Predicted Value (PPV)	0.90788477 [0.85298261, 0.96278693]
Negative Predicted Value (NPV)	0.99998505 [0.99986509, 1]
Positive Likelihood Ratio (PLR)	109.8749 [33.7796, 357.39]
Negative Likelihood ratio (NLR)	0.08854017 [0.04091139, 0.1916181]

**Table 5:** Egger’s Test.

Statistics	Value
Test statistics (t-value)	−0.4149
Degrees of Freedom (df)	13
*p*-value	0.685
Limit Estimate (b)	8.3678
Confidence Interval (CI)	(5.6490, 11.0866)

**Table 6:** Expanded Diagnostic Metrics for Subgroups.

Summary Table	All Neonates Population Studies [95% CI]	High Risk Population Studies (Without Eventov) [95% CI]	Seropositive Mothers Population Studies (Without Huang) [95% CI]
Total population	27,798	1536	283
Total Positives	167	268	45
Total Negatives	27,631	1268	238
Diagnostic Odds Ratio (DOR)	8635.7889097 [3197.511, 23323.41]	897.15102940 [351.83610399, 2287.6559865]	437.0323 [10.75779, 17,754.31]
Area Under the Curve (AUC)	0.9818733 [0.9575835, 1]	0.92594386 [0.86819714, 0.9836906]	0.9424269 [0.8681777, 1]
Sensitivity (SN)	0.9647973 [0.9163109, 1]	0.98101489 [0.93534775, 1]	0.9436141 [0.8409574, 1]
Specificity (SP)	0.9990369 [0.9986484, 0.9994253]	0.99567693 [0.99096294, 1]	0.9565622 [0.8629098, 1]
Positive Predicted Value (PPV)	0.8674074 [0.8092515, 0.9255633]	0.98170591 [0.95077107, 1]	0.8483736 [0.5857928, 1]
Negative Predicted Value (NPV)	0.9999896 [0.9998681, 1]	0.99402375 [0.98893884, 0.9991087]	0.9965186 [0.9816554, 1]
Positive Likelihood Ratio (PLR)	833.0070243 [517.1105, 1341.881]	29.49578286 [6.43471024, 135.2044108]	37.10282 [1.416866, 971.5942]
Negative Likelihood ratio (NLR)	0.1141102 [0.06201394, 0.2099710]	0.09141527 [0.03820145, 0.2187548]	0.08494893 [0.02705231, 0.2667543]

## Data Availability

The raw data table has been provided in Appendix D.

## Abbreviations

The following abbreviations are used in this manuscript:

CMV	Cytomegalovirus
SGA	Small for Gestational Age
PCR	Polymerase Chain Reaction
SN	Sensitivity
SP	Specificity
PPV	Positive Predicted Value
NPV	Negative Predicted Value
DOR	Diagnostic Odds Ratio
AUC	Area Under the Curve
PLR	Positive Likelihood Ratio
NLR	Negative Likelihood Ratio
SROC	Summary Receiver Operating Characteristic

## Appendix A. Search Strategies

PUBMED:

(newborns[MeSH Terms] OR newborn*[Title/Abstract] OR neonate*[Title/Abstract] OR infant*[Title/Abstract] OR babies[Title/Abstract] OR baby[Title/Abstract]) AND (cytomegalovirus[MeSH Terms] OR cytomegalovirus[Title/Abstract] OR “CMV”[Title/Abstract] OR “HCMV”[Title/Abstract] OR “Human Herpesvirus 5”[Title/Abstract] OR “HHV-5”[Title/Abstract]) AND (saliva[MeSH Terms] OR saliva*[Title/Abstract])

SCOPUS:

TITLE-ABS ((newborn* OR neonate* OR infant* OR babies OR baby) AND (cytomegalovirus OR “CMV” OR “HCMV” OR “Human Herpesvirus 5” OR “HHV-5”) AND (saliva OR saliva*))

Web Of Science:

TI = ((newborn* OR neonate* OR infant* OR babies OR baby) AND (cytomegalovirus OR “CMV” OR “HCMV” OR “Human Herpesvirus 5” OR “HHV-5”) AND (saliva OR saliva*)) OR AB = ((newborn* OR neonate* OR infant* OR babies OR baby) AND (cytomegalovirus OR “CMV” OR “HCMV” OR “Human Herpesvirus 5” OR “HHV-5”) AND (saliva OR saliva*))

ProQuest:

abstract(((newborn* OR neonate* OR infant* OR babies OR baby) AND (cytomegalovirus OR “CMV” OR “HCMV” OR “Human Herpesvirus 5” OR “HHV-5”) AND (saliva OR saliva*))) AND title(((newborn* OR neonate* OR infant* OR babies OR baby) AND (cytomegalovirus OR “CMV” OR “HCMV” OR “Human Herpesvirus 5” OR “HHV-5”) AND (saliva OR saliva*)))

Science direct:

Title, abstract, keywords ((Newborn OR neonate OR infant OR baby) AND (cytomegalovirus OR “CMV” OR “HCMV” OR “Human Herpesvirus”) AND (saliva OR salivary))

Cochrane library:

((Newborn OR neonate OR infant OR baby) AND (cytomegalovirus OR “CMV” OR “HCMV” OR “Human Herpesvirus”) AND (saliva OR salivary))

CINAHL:

TI (((newborn* OR neonate* OR infant* OR babies OR baby) AND (cytomegalovirus OR “CMV” OR “HCMV” OR “Human Herpesvirus 5” OR “HHV-5”) AND (saliva OR saliva*))) AND AB (((newborn* OR neonate* OR infant* OR babies OR baby) AND (cytomegalovirus OR “CMV” OR “HCMV” OR “Human Herpesvirus 5” OR “HHV-5”) AND (saliva OR saliva*)))

Wiley’s online library:

“((newborn* OR neonate* OR infant* OR babies OR baby) AND (cytomegalovirus OR “CMV” OR “HCMV” OR “Human Herpesvirus 5” OR “HHV-5”) AND (saliva OR saliva*))” in Title and “((newborn* OR neonate* OR infant* OR babies OR baby) AND (cytomegalovirus OR “CMV” OR “HCMV” OR “Human Herpesvirus 5” OR “HHV-5”) AND (saliva OR saliva*))” in Abstract

## Appendix B. Quality Assessment Template

**Table A1:** QUDAS-2 Survey.

QUDAS-2 Survey
***Patient Selection***
Was a case–control design avoided?
Were inappropriate exclusions avoided?
**Risk of Bias:** Was patient selection conducted in a way that avoids bias?
**Applicability Concern:** Do the included patients match the review question?
***Index Test***
Was the index test (test being evaluated) result interpreted without knowledge of the reference standard? (Blinded interpretation)
Was the test threshold pre-specified (rather than determined post hoc)?
**Risk of Bias:** Was the index test conducted in a way that avoids bias?
**Applicability Concern:** Does the index test match the review question?
***Reference Standard***
Is the reference standard likely to correctly classify the target condition?
Was the reference standard interpreted without knowledge of the index test? (Blinded interpretation)
**Risk of Bias:** Was the reference standard appropriate?
**Applicability Concern:** Does the reference standard match the review question?
***Flow and Timing***
Was there an appropriate time interval between the index test and reference standard?
Did all patients receive the same reference standard?
Were all patients included in the final analysis?
**Risk of Bias:** Was patient flow and timing handled appropriately?

## Appendix D. Raw Data Table

**Table A2:** Raw Data Table for meta-analysis [17,25,26,27,28,29,30,31,32,33,34,35,36,37,38].

Study ID	Total Number of Participants	TP	FP	TN	FN	SP	Standard Error	Lower CI	Upper CI	SN	Standard Error 2	Lower CI 2	Upper CI 2	PPV	Standard Error 3	Lower CI 3	Upper CI 3	NPV	Standard Error 4	Lower CI 4	Upper CI 4
Alkhawaja 2012 [25]	150	8	0	142	0	1	0.006901408	0.973659093	1	1	0.1225	0.67558438	1	1	0.1225	0.67558438	1	1	0.006901408	0.973659093	1
Barkai 2014 [26]	9845	48	8	9789	0	0.9992	0.000302652	0.998411364	0.999597297	1	0.020416667	0.925897349	1	0.8571	0.049933812	0.74258818	0.925762933	1	0.000100112	0.999607713	1
Cardoso 2015 [27]	333	4	0	328	1	1	0.002987805	0.988423392	1	0.8	0.265360133	0.375528264	0.963776839	1	0.245	0.51009998	1	0.997	0.004238393	0.983052342	0.999475082
Choudhary 2015 [28]	18	18	0	0	0	-	-	-	-	1	0.054444444	0.824115449	1	1	0.054444444	0.824115449	1	-	-	-	-
Dollard 2021 [17]	12,554	52	8	12,490	4	0.999	0.000293396	0.998271786	0.999421546	0.929	0.038523879	0.830800296	0.972119412	0.867	0.046782772	0.758739536	0.931092799	1	7.84377 E-05	0.999692619	1
Eventov-Friedman 2019 [29]	856	57	68	730	1	0.915	0.009948395	0.893606311	0.932417183	0.983	0.988489251	0.199595396	0.999925424	0.456	0.062094275	0.343155986	0.573549656	0.999	0.001778738	0.992923231	0.999859431
Exler 2019 [30]	133	27	10	94	2	0.904	0.030385127	0.832175114	0.947041811	0.931	0.057940588	0.780304765	0.980863997	0.73	0.077643808	0.570498487	0.846233472	0.98	0.017560659	0.928436508	0.994625623
Fernandes 2021 [31]	1492	10	4	1478	0	0.9973	0.001501406	0.993079098	0.998949392	1	0.098	0.722459831	1	0.7143	0.139559386	0.453518208	0.882796921	1	0.000663058	0.99740755	1
Huang 2021 [33]	6234	12	0	6185	37	1	0.000158448	0.99937927	1	0.245	0.06461424	0.146101715	0.380975436	1	0.081666667	0.757499243	1	0.994	0.000991636	0.991752774	0.995637589
Izquierdo 2023 [34]	1651	7	2	1641	1	0.998	0.001253247	0.994387678	0.999288945	0.875	1.03429928	0.158743682	0.996163786	0.777	0.57160782	0.211260159	0.978413903	0.999	0.000982148	0.995914755	0.999755788
Izquierdo 2023.2 [34]	119	2	0	116	1	1	0.008448276	0.967944353	1	0.666	0.425275336	0.207287919	0.938292062	1	0.49	0.342371953	1	0.9914	0.011959543	0.953082694	0.998473723
Izquierdo 2024 [34]	190	3	0	186	1	1	0.005268817	0.979764182	1	0.75	0.326955654	0.300636052	0.954413937	1	0.326666667	0.43849392	1	0.995	0.007353136	0.970913725	0.99915779
Pasternak 2020 [35]	83	42	0	41	0	1	0.023902439	0.914329551	1	1	-	-	-	1	-	-	-	1	0.023902439	0.914329551	1
Pasternak 2020.2 [35]	83	41	0	41	1	1	0.023902439	0.914329551	1	0.976	0.991879025	0.196777328	0.999851885	1	-	-	-	0.976	0.033198776	0.876493769	0.995727096
Pasternak 2020.3 [35]	83	42	3	38	0	0.927	0.047136385	0.805946267	0.97489117	1	-	-	-	0.933	0.357138784	0.382925624	0.99681011	1	0.025789474	0.908187067	1
Ross 2014 [36]	80	79	1	0	0	0	0.98	0	0.793456709	1	0.012405063	0.953627163	1	0.988	0.017270278	0.93334121	0.997938727	-	-	-	-
Stark 2024 [37]	880	33	4	838	5	0.994	0.002904787	0.986088851	0.997423901	0.868	0.060665104	0.726226125	0.942199556	0.892	0.057490953	0.753044839	0.95721137	0.994	0.002902787	0.986095372	0.997422679
Yamamoto 2006 [38]	1923	22	2	1895	4	0.999	0.000890787	0.996249107	0.999733936	0.846	0.080197463	0.664506503	0.938410231	0.917	0.069560425	0.741935722	0.976988478	0.998	0.00114778	0.994749493	0.999239707

## Appendix E. Study Characteristics Table

**Table A3:** Study Characteristics Table.

Study	Year of Publication	Country in Which the Study Conducted	Total Population	Temporal Classification	Study Design	Type of Test Conducted for Saliva	Type of Test Conducted for Urine	Saliva Sample	Urine Sample	Population Type	Age of Children	SP	SN	P PV	NPV
Alkhawaja 2012 [25]	2012	Bahrain	150	prospective	cohort	nested PCR	nested PCR	Liquid	unclear	seropositive mothers	<6 wks	1.000	1.000	1.000	1.000
Barkai 2014 [26]	2014	Israel	9845	prospective	cross-sectional	RT-PCR	RT-PCR	unclear	unclear	All	<4 wks	0.999	1.000	0.857	1.000
Cardoso 2015 [27]	2015	Brazil	333	prospective	cross-sectional	nested PCR	nested PCR	Liquid	Bag	All	<3 wks	1.000	0.800	1.000	0.997
Choudhary 2015 [28]	2015	India	18	prospective	cohort	PCR	PCR	Liquid	Sterile tube	High-risk	<10 months	-	1.000	1.000	-
Dollard 2021 [17]	2021	United States	12,554	prospective	cohort	PCR	PCR	Dry	unclear	All	<3 wks	0.999	0.929	0.867	1.000
Eventov-Friedman 2019 [29]	2019	Israel	856	prospective	cohort	PCR	PCR	Liquid	Bag	High-risk	<10 days	0.915	0.983	0.456	0.999
Exler 2019 [30]	2019	Germany	133	retrospective	cohort	PCR	PCR	Liquid	unclear	seropositive mothers	<2 wks	0.904	0.931	0.730	0.980
Fernandes 2021 [31]	2021	Portugal	1492	prospective	cross-sectional	qPCR	qPCR	Liquid	Bag	All	<2 wks	0.997	1.000	0.714	1.000
Huang 2021 [33]	2021	China	6234	prospective	cohort	qPCR	qPCR	Liquid	Bag	seropositive mothers	<3 wks	1.000	0.245	1.000	0.994
Izquierdo 2023 [34]	2023	Chile	1651	prospective	cross-sectional	RT-PCR	RT-PCR	Dry	Bag	All	<3 wks	0.998	0.875	0.777	0.999
**Izquierdo 2023.2** [34]	**2023**	**Chile**	**119**	**prospective**	**cross-sectional**	**RT-PCR**	**RT-PCR**	**Dry**	**Bag**	**High-risk**	**<3 wks**	**1.000**	**0.666**	**1.000**	**0.991**
Izquierdo 2024 [34]	2024	Chile	190	prospective	cross-sectional	RT-PCR	RT-PCR	Dry	Bag	High-risk	<3 wks	1.000	0.750	1.000	0.995
Pasternak 2020 [35]	2020	Israel	83	prospective	case–control	qPCR	qPCR	Liquid	Bag	High-risk	<4 wks	1.000	1.000	1.000	1.000
**Pasternak 2020.2** [35]	**2020**	**Israel**	**83**	**prospective**	**case–control**	**qPCR**	**qPCR**	**Wet**	**Bag**	**High-risk**	**<4 wks**	**1.000**	**0.976**	**1.000**	**0.976**
**Pasternak 2020.3** [35]	**2020**	**Israel**	**83**	**prospective**	**case–control**	**qPCR**	**qPCR**	**Dry**	**Bag**	**High-risk**	**<4 wks**	**0.927**	**1.000**	**0.933**	**1.000**
Ross 2014 [36]	2014	United States	80	prospective	cohort	qPCR	qPCR	Liquid	Bag	High-risk	<3 wks	0.000	1.000	0.988	-
Stark 2024 [37]	2024	United States	880	retrospective	cohort	qPCR	PCR	unclear	unclear	High-risk	<3 wks	0.994	0.868	0.892	0.994
Yamamoto 2006 [38]	2006	Brazil	1923	prospective	cross-sectional	PCR	PCR	Liquid	Sterile tube	All	<3 wks	0.999	0.846	0.917	0.998

## Appendix F. Full LOO Tables

**Table A4:** Leave-Out-Analysis 1-Specificity.

Study	Estimate	SE	Z_Value	*p*_Value	CI_Lower	CI_Upper	Q	Qp	Tau^2^	I^2^	H^2^
Alkhawaja 2012 [25]	0.99835	0.00068	1473.69292	<0.0001	0.99702	0.99968	105.06197	5.04392E-16	2.48461E-06	86.67453	7.50443
Barkai 2014 [26]	0.99790	0.00088	1128.29218	<0.0001	0.99616	0.99963	102.93035	1.29820E-15	3.99736E-06	86.39857	7.35217
Cardoso 2015 [27]	0.99830	0.00069	1448.59914	<0.0001	0.99695	0.99965	105.04763	5.07616E-16	2.49311E-06	86.67271	7.50340
Dollard 2021 [17]	0.99793	0.00089	1126.86722	<0.0001	0.99620	0.99967	99.91582	4.92191E-15	4.01000E-06	85.98820	7.13684
**Eventov-Friedman 2019** [29]	**0.99908**	**0.00038**	**2662.53506**	**<0.0001**	**0.99835**	**0.99982**	**32.73220**	**3.14837E-03**	**5.10888E-07**	**57.22866**	**2.33801**
Exler 2019 [30]	0.99847	0.00065	1547.15384	<0.0001	0.99720	0.99973	95.16536	3.97717E-14	2.21286E-06	85.28876	6.79753
Fernandes 2021 [31]	0.99849	0.00071	1413.18368	<0.0001	0.99710	0.99987	102.69548	1.44050E-15	2.46067E-06	86.36746	7.33539
Huang 2021 [33]	0.99759	0.00096	1037.24469	<0.0001	0.99570	0.99947	88.89298	6.13968E-13	4.98788E-06	84.25072	6.34950
Izquierdo 2023 [34]	0.99841	0.00072	1391.73049	<0.0001	0.99700	0.99982	103.41226	1.04860E-15	2.49966E-06	86.46195	7.38659
Izquierdo 2023.2 [34]	0.99836	0.00068	1476.01648	<0.0001	0.99703	0.99968	105.06307	5.04146E-16	2.48396E-06	86.67467	7.50451
Izquierdo 2024 [34]	0.99834	0.00068	1468.91767	<0.0001	0.99701	0.99967	105.05961	5.04923E-16	2.48601E-06	86.67423	7.50426
Pasternak 2020 [35]	0.99837	0.00067	1480.26758	<0.0001	0.99704	0.99969	105.06500	5.03715E-16	2.48282E-06	86.67492	7.50464
Pasternak 2020.2 [35]	0.99837	0.00067	1480.26758	<0.0001	0.99704	0.99969	105.06500	5.03715E-16	2.48282E-06	86.67492	7.50464
Pasternak 2020.3 [35]	0.99839	0.00067	1495.79361	<0.0001	0.99709	0.99970	102.69279	1.44222E-15	2.41803E-06	86.36711	7.33520
Stark 2024 [37]	0.99857	0.00068	1471.41615	<0.0001	0.99724	0.99990	101.33737	2.62705E-15	2.39212E-06	86.18476	7.23838
Yamamoto 2006 [38]	0.99824	0.00074	1350.65444	<0.0001	0.99679	0.99969	104.59739	6.19929E-16	2.59703E-06	86.61534	7.47124

**Table A5:** Leave-Out-Analysis 2-Sensitivity.

Study	Estimate	SE	Z_Value	*p*_Value	CI_Lower	CI_Upper	Q	Qp	Tau^2^	I^2^	H^2^
Alkhawaja 2012 [25]	0.86748	0.04870	17.81205	<0.0001	0.77202	0.96293	142.47142	2.28664E-23	0.01954	90.17347	10.17653
Barkai 2014 [26]	0.85824	0.06241	13.75202	<0.0001	0.73592	0.98056	140.02984	6.99861E-23	0.03393	90.00213	10.00213
Cardoso 2015 [27]	0.87799	0.04743	18.51029	<0.0001	0.78502	0.97096	142.10809	2.70109E-23	0.01927	90.14834	10.15058
Choudhary 2015 [28]	0.86208	0.05076	16.98425	<0.0001	0.76260	0.96156	142.24243	2.53976E-23	0.02067	90.15765	10.16017
Dollard 2021 [17]	0.86898	0.05233	16.60448	<0.0001	0.76640	0.97155	141.23326	4.03325E-23	0.02211	90.08732	10.08809
Eventov-Friedman 2019 [29]	0.87584	0.04691	18.67088	<0.0001	0.78390	0.96778	142.52594	2.23019E-23	0.01928	90.17723	10.18042
Exler 2019 [30]	0.86978	0.05050	17.22255	<0.0001	0.77080	0.96876	142.02524	2.80564E-23	0.02046	90.14260	10.14466
Fernandes 2021 [31]	0.86571	0.04917	17.60662	<0.0001	0.76934	0.96208	142.44039	2.31941E-23	0.01969	90.17133	10.17431
**Huang 2021** [33]	**0.98751**	**0.00961**	**102.79747**	**<0.0001**	**0.96868**	**1.00633**	**13.33019**	**5.00705E-01**	**0.00000**	**0.00000**	**1.00000**
Izquierdo 2023 [34]	0.87608	0.04690	18.67837	<0.0001	0.78415	0.96801	142.51738	2.23896E-23	0.01927	90.17664	10.17981
Izquierdo 2023.2 [34]	0.87842	0.04706	18.66679	<0.0001	0.78619	0.97066	142.00995	2.82538E-23	0.01922	90.14154	10.14357
Izquierdo 2024 [34]	0.87832	0.04724	18.59445	<0.0001	0.78574	0.97090	142.06694	2.75252E-23	0.01924	90.14549	10.14764
Pasternak 2020.2 [35]	0.87586	0.04691	18.67139	<0.0001	0.78392	0.96780	142.52605	2.23007E-23	0.01928	90.17723	10.18043
Ross 2014 [36]	0.85778	0.06461	13.27719	<0.0001	0.73116	0.98440	129.70910	7.75839E-21	0.03686	89.20662	9.26494
Stark 2024 [37]	0.87669	0.04996	17.54918	<0.0001	0.77878	0.97460	139.54507	8.73733E-23	0.01995	89.96740	9.96751
Yamamoto 2006 [38]	0.87880	0.04926	17.84000	<0.0001	0.78225	0.97535	140.04435	6.95228E-23	0.01953	90.00317	10.00317

**Table A6:** Leave-Out-Analysis 3-PPV.

Study	Estimate	SE	Z_Value	*p*_Value	CI_Lower	CI_Upper	Q	Qp	Tau^2^	I^2^	H^2^
Alkhawaja 2012 [25]	0.84579	0.04740	17.84378	<0.0001	0.75289	0.93869	93.34868	8.81038E-14	0.01986	85.00247	6.66776
Barkai 2014 [26]	0.85437	0.04999	17.08923	<0.0001	0.75639	0.95236	90.07972	3.66575E-13	0.02176	84.45821	6.43427
Cardoso 2015 [27]	0.85655	0.04610	18.57963	<0.0001	0.76619	0.94691	93.22106	9.31590E-14	0.01944	84.98194	6.65865
Choudhary 2015 [28]	0.83993	0.05006	16.77826	<0.0001	0.74182	0.93805	92.57471	1.23559E-13	0.02189	84.87708	6.61248
Dollard 2021 [17]	0.85318	0.05056	16.87489	<0.0001	0.75408	0.95227	90.39569	3.19487E-13	0.02237	84.51254	6.45684
**Eventov-Friedman 2019** [29]	**0.90788**	**0.02801**	**32.41077**	**<0.0001**	**0.85298**	**0.96279**	**28.86187**	**1.09092E-02**	**0.00401**	**51.49310**	**2.06156**
Exler 2019 [30]	0.86662	0.04674	18.54056	<0.0001	0.77501	0.95824	85.50974	2.65604E-12	0.01859	83.62760	6.10784
Fernandes 2021 [31]	0.86325	0.04650	18.56453	<0.0001	0.77211	0.95439	90.71594	2.77911E-13	0.01909	84.56721	6.47971
Huang 2021 [33]	0.84242	0.04843	17.39460	<0.0001	0.74750	0.93734	93.11580	9.75457E-14	0.02044	84.96496	6.65113
Izquierdo 2023 [34]	0.85572	0.04568	18.73342	<0.0001	0.76619	0.94525	93.44245	8.45646E-14	0.01941	85.01752	6.67446
Izquierdo 2023.2 [34]	0.85725	0.04572	18.75095	<0.0001	0.76764	0.94685	93.09243	9.85470E-14	0.01934	84.96118	6.64946
Izquierdo 2024 [34]	0.85702	0.04590	18.67118	<0.0001	0.76705	0.94698	93.16475	9.54805E-14	0.01939	84.97286	6.65463
Pasternak 2020.3 [35]	0.85412	0.04592	18.60090	<0.0001	0.76412	0.94412	93.53004	8.13867E-14	0.01947	85.03155	6.68072
Ross 2014 [36]	0.83938	0.04929	17.02881	<0.0001	0.74277	0.93598	58.00863	2.61361E-07	0.02065	75.86566	4.14347
Stark 2024 [37]	0.85096	0.04975	17.10584	<0.0001	0.75346	0.94846	92.55589	1.24578E-13	0.02157	84.87400	6.61114
Yamamoto 2006 [38]	0.84903	0.04905	17.30819	<0.0001	0.75289	0.94518	93.33104	8.87861E-14	0.02096	84.99963	6.66650

**Table A7:** Leave-Out-Analysis 4-NPV.

Study	Estimate	SE	Z_Value	*p*_Value	CI_Lower	CI_Upper	Q	Qp	Tau^2^	I^2^	H^2^
Alkhawaja 2012 [25]	0.99929	2.926E-04	3415.01153	<0.0001	0.99872	0.99986	48.19143	1.22202E-05	2.58765E-07	70.94919	3.44224
Barkai 2014 [26]	0.99807	7.727E-04	1291.64409	<0.0001	0.99655	0.99958	47.96598	1.33172E-05	3.08023E-06	70.81265	3.42614
Cardoso 2015 [27]	0.99931	2.916E-04	3427.09513	<0.0001	0.99873	0.99988	47.70285	1.47204E-05	2.55168E-07	70.65165	3.40735
Dollard 2021 [17]	0.99807	7.734E-04	1290.47523	<0.0001	0.99655	0.99958	47.60547	1.52758E-05	3.08688E-06	70.59162	3.40039
Eventov-Friedman 2019 [29]	0.99930	2.955E-04	3381.54984	<0.0001	0.99872	0.99988	47.89841	1.36645E-05	2.57413E-07	70.77147	3.42131
Exler 2019 [30]	0.99931	2.884E-04	3464.84240	<0.0001	0.99874	0.99988	46.89921	1.99691E-05	2.48935E-07	70.14875	3.34994
Fernandes 2021 [31]	0.99918	3.151E-04	3170.71180	<0.0001	0.99856	0.99980	48.18820	1.22353E-05	2.65545E-07	70.94725	3.44201
**Huang 2021** [33]	**0.99999**	**6.121E-05**	**16337.66443**	**<0.0001**	**0.99987**	**1.00011**	**11.90201**	**6.14172E-01**	**0.00000**	**0.00000**	**1.00000**
Izquierdo 2023 [34]	0.99932	3.012E-04	3317.47315	<0.0001	0.99873	0.99991	47.22766	1.76324E-05	2.54414E-07	70.35635	3.37340
Izquierdo 2023.2 [34]	0.99930	2.909E-04	3435.58932	<0.0001	0.99873	0.99987	47.67887	1.48553E-05	2.54845E-07	70.63689	3.40563
Izquierdo 2024 [34]	0.99930	2.912E-04	3431.71177	<0.0001	0.99873	0.99987	47.73599	1.45360E-05	2.55311E-07	70.67202	3.40971
Pasternak 2020 [35]	0.99929	2.924E-04	3418.07605	<0.0001	0.99872	0.99986	48.19146	1.22201E-05	2.58709E-07	70.94921	3.44225
Pasternak 2020.2 [35]	0.99930	2.908E-04	3436.84941	<0.0001	0.99873	0.99987	47.67048	1.49028E-05	2.54764E-07	70.63172	3.40503
Pasternak 2020.3 [35]	0.99929	2.924E-04	3418.11551	<0.0001	0.99872	0.99986	48.19146	1.22201E-05	2.58708E-07	70.94921	3.44225
Stark 2024 [37]	0.99939	2.804E-04	3564.54656	<0.0001	0.99884	0.99994	43.97065	5.98460E-05	2.27075E-07	68.16058	3.14076
Yamamoto 2006 [38]	0.99939	2.917E-04	3425.77611	<0.0001	0.99882	0.99996	45.26014	3.70113E-05	2.38587E-07	69.06770	3.23287
